# Iron accumulation dynamics in *Nostoc* sp. PCC 7120: extent of biosorption in batch and continuous reactors

**DOI:** 10.1186/s13068-026-02743-9

**Published:** 2026-01-28

**Authors:** Veronica Lucato, Giulia Mazzola, Fengzheng Gao, Fabian Abiusi, Denis Badocco, Paolo Pastore, Alexander Mathys, Eleonora Sforza

**Affiliations:** 1 Department of Industrial Engineering DII, University of Padova, Via Marzolo 9, 35131 Padua, Italy; 2 Institute of Food, Nutrition and Health, Sustainable Food Processing, ETH Zurich, 8092 Zurich, Switzerland; 3 Institute of Food, Nutrition and Health, Laboratory of Nutrition and Metabolic Epigenetics, ETH Zurich, 8603 Schwerzenbach, Switzerland; 4 Department of Inorganic, Metallorganic and Analytical Chemistry, University of Padua, Via Marzolo 1, 35131 Padua, Italy

**Keywords:** Biosorption phenomena, Iron uptake, Internal Quota, Iron adsorption, Exopolysaccharides

## Abstract

**Background:**

Microalgae have recently gained attention for their ability to incorporate iron into organic molecules, potentially enhancing bioavailability and mitigating associated risks, suggesting a promising role in the field of iron supplementation. Among microalgal species, nitrogen-fixing organisms present a relatively high iron demand due to their unique metabolic requirements, positioning them as promising candidates for addressing iron deficiencies. However, information on the dynamics and extent of iron accumulation in diazotrophic cyanobacteria remains limited. Understanding how these organisms regulate iron uptake and storage under different cultivation conditions is essential to evaluate their potential for nutritional and biotechnological applications.

**Results:**

This work investigates the iron accumulation dynamics of the diazotrophic species *Nostoc* sp. PCC 7120 under both batch and continuous systems. The results highlight its ability to adapt and survive across a wide range of iron concentration and demonstrate a strong correlation between iron availability and the internal quota of iron in biomass. High-density batch cultivation revealed that the iron content in *Nostoc* sp. can span a remarkably wide range, up to tens of thousands of mg_Fe_ kg_X_^−1^, far exceeding typical values reported in the literature. This high accumulation mainly results from consistent external biosorption of the metal onto the cell surface. In continuous steady-state culture, the externally biosorbed fraction was lower but still detectable, suggesting that adsorption substantially contributes to overall iron accumulation. Under strong iron limitation (below 0.5 mg_Fe_ L^−1^), a marked increase in exopolysaccharide production was observed, suggesting that the extracellular matrix plays a functional role in the biosorption of the metal.

**Conclusions:**

*Nostoc* sp. PCC 7120 exhibits an exceptional capacity to tolerate and accumulate iron over a broad concentration range, mediated by both intracellular uptake and extracellular adsorption. These findings reveal that diazotrophic cyanobacteria can effectively modulate their iron management strategies under different regimes and highlight their potential as biofactories for iron-enriched biomass and as model systems for studying biologically driven metal sequestration.

**Supplementary Information:**

The online version contains supplementary material available at 10.1186/s13068-026-02743-9.

## Background

Microalgae and cyanobacteria are increasingly proposed as functional food sources because of their compelling ability to accumulate biomolecules and minerals that are beneficial for health [1]. Among these minerals, iron is particularly important in nutraceutical applications [2]. Untreated iron deficiency, which affects more than 25% of the global population, leads to severe chronic health issues [3]. One of the main challenges in addressing iron deficiencies lies in the bioavailability of iron [4]. *Heme* iron, which is found exclusively in animal-derived foods, is up to ten times more bioavailable than *non-heme* iron from plants is [5], but meat consumption is associated with environmental sustainability issues [6] and may not be feasible in developing countries [7]. On the other hand, although the World Health Organisation recommends the use of compounds such as ferrous sulfate, ferrous fumarate, ferric pyrophosphate, and electrolytic iron powder, many foods are fortified with non-recommended sources with lower bioavailability, limiting the effectiveness of this approach [8].

Microalgae are recognised for their ability to accumulate iron, with reported concentrations ranging from 200 to 2000 mg per kg of biomass [9]. Iron in microalgae is primarily *non-heme*, but its bioavailability is often greater than that in many conventional vegetal-based sources, as the latter contain greater levels of absorption inhibitors such as phytates, polyphenols, and tannins [8]. Moreover, microalgae offer a rich nutritional profile that includes vitamins and antioxidants, such as vitamins A and C, and β-carotene, which are known to increase iron adsorption [10, 11].

In microalgae, iron is essential for several biochemical reactions because of its redox properties, which make it both indispensable and potentially toxic because of the generation of reactive oxygen species (ROS) [12]. To mitigate the risk of toxicity, iron homeostasis is tightly regulated. It is rarely found in its free ionic form and is typically stored as ferric iron (Fe^3+^) in proteins, such as ferritins [13]. When biologically active, iron primarily functions in its ferrous form (Fe^2+^) [14]. Photosynthetic microorganisms, including cyanobacteria, have particularly high iron requirements because of their Fe-rich photosynthetic apparatus. Iron is involved in the reaction centers of photosystems (PSs), with 2–3 atoms in PSII and up to 12 in PSI [15, 16]. It also plays key roles in molecules such as ferredoxin, cytochrome C_6_, and the cytochrome b_6_f complex [17]. In addition, iron is a core component of superoxide dismutase (SOD), an enzyme responsible for detoxifying ROS. This function is particularly important in nitrogen-fixing cyanobacteria, where SOD activity mitigates oxidative stress during heterocyst formation, supporting proper cellular differentiation [18]. Another critical role of iron is in nitrogenase metal centers, where it serves as an active site for nitrogen fixation [19]. As nitrogen-fixing cyanobacteria rely on iron for both photosynthesis and nitrogen fixation, they are estimated to require 2.5 to 5.2 times more iron than their non-N-fixing counterparts [20].

To date, research efforts on cyanobacteria and iron have focused mainly on their role in bioremediation, particularly in removing heavy metals from liquid streams [21–23]. In contrast, far less attention has been given to their potential in biologically complexing iron with organic molecules for nutraceutical and functional food applications. The existing data on the iron content in cyanobacteria are restricted to a limited number of genera, such as *Arthrospira*, *Chlorella*, *Nannochloropsis*, and *Phaeodactylum*, and the observed ranges (i.e., 200–2000 mg_Fe_ kg_X_^−1^) vary significantly depending on the species and cultivation conditions [24–27]. Most research has focused on understanding cyanobacterial responses under limited conditions, with few studies exploring the effect of iron abundance [28, 29]. There has been little exploration of how different cultivation systems and operating processes influence the biomass iron content, with most studies relying on batch cultivation systems, which provide valuable insights into short-term physiological responses but fail to capture the results of acclimation.

This study addresses this gap by focusing on *Nostoc* sp. PCC 7120, a nitrogen-fixing cyanobacterium that has already demonstrated a favourable composition for use as an alternative food source, particularly because of its protein content, amino acid profile, and protein bioaccessibility [30]. To expand its potential applications in functional foods, this work delves into its ability to accumulate iron. Specifically, the effects of different iron concentration on *Nostoc* sp. PCC 7120 biomass growth kinetics, composition, and iron uptake dynamics within a high-density system were investigated. Additionally, continuous cultivation was employed to evaluate the steady-state responses to different iron concentrations and residence time, examining their impact on the biomass concentration and composition along with the iron distribution between the intracellular and adsorbed fractions. The novelty of this work lies in its quantitative analysis of iron quota in both transient and steady-state cultures, highlighting the kinetics and the allocation of iron within the cells.

## Methods

### Cyanobacterial strains and culture conditions

*Nostoc* sp. PCC 7120, obtained from the UTEX collection (Austin, USA), was maintained axenically in 250-mL Drechsel® bottles with a 5-cm diameter. The cultures were maintained at 27 ± 1 °C in a temperature-controlled incubator under continuous illumination at an intensity of 300 µmol m^−2^ s^−1^, provided by a photosynthetically active radiation (PAR)-emitting lamp. Mixing was ensured by magnetic stirring at the bottom of the culture and bubbling with 5% CO_2_-enriched air (v/v), which served as both an inorganic and dinitrogen source. The strain was cultivated under diazo-phototrophic conditions in BG11_0_ media devoid of any combined nitrogen sources. The composition of the BG11_0_ medium included the following components at the given concentration (mg L^−1^): 1.0 Na_2_Mg EDTA, 6.215 FeCl_3_·6 H_2_O, 6 citric acid·1H_2_O, 36 CaCl_2_·2 H_2_O, 75 MgSO_4_·7 H_2_O, 2.86 H_3_BO_3_, 1.81 MnCl_2_·4 H_2_O, 0.222 ZnSO_4_·7 H_2_O, 0.079 CuSO_4_·5 H_2_O, 0.391 NaMoO_4_·2 H_2_O, 0.05 CoCl_2_·6 H_2_O, and 250 NaHCO_3_.

Batch experiments were performed within a high-density (HD) cultivation system (CellDEG HD100 Cultivator) over 7–8 days, during which the biomass concentration (*C*_*X*_, g_X_ L^−1^), iron depletion from the culture medium (*C*_*Fe*_, mg_Fe_ L^−1^), and iron accumulation in the biomass (*q*_*Fe*_, mg_Fe_ kg_X_^−1^) were monitored. The specific growth rate (*µ*, d^−1^) during the exponential growth phase was also evaluated. A description of the HD system is provided by Lippi et al. [31]. The cultivation conditions were determined on the basis of previous experimental results to ensure optimal *Nostoc* sp. growth within the system and to highlight the effects of iron concentration in the media (*C*_*Fe,0*_). The experiments were carried out at 27 ± 1 °C [32]. Light intensity, orbital shaking, and nutrient concentrations were set according to optimised conditions established through a design of experiments (DoE) approach described in a previous study [33]. The conditions applied included continuous illumination at 480 ± 5 µmol m^−2^ s^−1^ provided by a white LED lamp (LED KE 308, DH Licht, Germany), orbital shaking at 110 ± 10 rpm, and 1.5% CO_2_-enriched air. Nutrient concentrations in the BG11_0_ medium were increased fivefold (fivefold) compared with those in the standard formulation to support high cell densities and avoid nutrient limitations that could interfere with the effects of iron concentration. Exceptions were made for iron and phosphorus, with phosphorus maintained at 34 mg_P_ L^−1^ to prevent growth inhibition [33], and the iron concentration (*C*_*Fe,0*_) ranged from 0 to 12.0 mg_Fe_ L^−1^. The effects of different culture pretreatments were also evaluated. Precultures were grown in iron-free BG11_0_ (fivefold) medium to deplete biomass iron reserves, with two pretreatment periods applied: 2 days and 10 days, based on preliminary observation on internal iron quota evolution (data not shown). Specifically, the following *C*_*Fe,0*_ were tested: 0.00, 0.45, 0.80, 2.00, 4.50 and 8.50 mg_Fe_ L^−1^ after 2 days of pretreatment and 0, 0.3, 0.7, 1.3, 5.0 and 12.0 mg_Fe_ L^−1^ after 10 days of deprivation. The choice of the iron concentration range for HD cultivators was based on previous results [33], by considering the expected biomass final concentration obtainable, which is particularly high in such a system.

Continuous experiments (chemostats) were performed in flat-panel photobioreactors (PBRs) with a working volume of 200 mL and a thickness of 3.5 cm. The PBRs featured transparent surfaces to facilitate light penetration and were free of metal components to prevent interference with iron in the culture environment. The system was approximated as a continuous stirred-tank reactor (CSTR) due to effective mixing provided by magnetic stirring at the reactor base and bubbling of 5% (v/v) CO_2_-enriched air. The culture volume was kept constant through a level control system, while the residence time, or hydraulic retention time (HRT), was adjusted by varying the inflow rate of fresh medium, supplied continuously by a peristaltic pump (205S/CA, Watson Marlow Fluid Technology Group). The temperature was maintained at 24 ± 1 °C within a temperature-controlled incubator, and the light was continuously provided by an LED lamp (Panda Grow XMSJ CXB3590-X1) at an intensity of 550 µmol m^−2^ s^−1^ to achieve light saturation levels [30]. The effects of different iron concentration (*C*_*Fe,in*_) and residence times (*τ*) were evaluated. The values of C_Fe,in_ were 0.17, 0.48, 1.30, and 2.85 mg_Fe_ L^−1^, whereas the values of τ were 1.2 ± 0.1 d and 2.2 ± 0.1 d. The range of iron concentration and residence times were selected based on previous results of continuous cultivation [30] and steady-state values of biomass concentrations expected.

Continuous experiments were monitored until a steady state was reached, at which pH, biomass concentration and composition were measured. The steady state was considered to have been reached after at least three HRTs had elapsed. For each HRT used, four consecutive HRT cycles were monitored and considered biological replicates (n = 4). Analyses also included the quantification of nitrogen, carbohydrates, and pigments (chlorophyll *a*, total carotenoids, and phycobiliproteins) in the biomass and the production of exopolysaccharides (EPS). Additionally, the iron concentration in the culture medium (*C*_*Fe*_, mg_Fe_ L^−1^) and its accumulation in the biomass (*q*_*Fe*_, mg_Fe_ kg_X_^−1^) were evaluated.

The pH was measured daily via a portable pH probe (Hanna HI9124), and the light intensity was monitored as the photon flux density (PFD) with a photoradiometer (HD 2101.1 from Delta OHM). All culture media were sterilised by autoclaving for 20 min at 121 °C. To prevent salt precipitation, sodium bicarbonate and FeCl_3_ EDTA were added afterwards, following proper sterilisation.

All experiments were performed in metal-free, non-glass photobioreactors, which were cleaned with 11% (v/v) H_2_O_2_ and subsequently rinsed with ultrapure water (18.2 MΩ·cm, Milli-Q grade) prior to inoculation to minimise potential iron contamination.

### Biomass growth and composition analyses

The biomass concentration was assessed by measuring the optical density (OD) at 750 nm via a dual-beam spectrometer (UV 1900, Shimadzu, Japan) and the dry weight (DW) via vacuum filtration of 10-mL culture samples on pre-dried 0.45 µm nitrocellulose filters, followed by drying at 105 °C (2 h) and gravimetric analysis. The biomass concentration (*C*_*X*_, g_X_ L^−1^) was calculated from the sample volume, gross weight, and filter tare weight. The supernatants were stored at −18 °C for subsequent analyses.

The growth rate (*µ*, d^−1^) was calculated from the slope of ln(OD) over time during the exponential growth phase in batch experiments and as the inverse of the residence time in continuous reactors.

The growth rate as a function of iron concentration was fitted according to the Monod equation via the Excel solver.

1 $$\mu ={\mu }_{max}\frac{{C}_{Fe,0}}{{k}_{Fe}+{C}_{Fe,0}}$$

The carbohydrate content in the biomass was quantified via the anthrone colorimetric method [34] and expressed as a percentage of the biomass dry weight (% DW). The primary pigments, chlorophyll *a* (Chl*a*) and total carotenoids (TCs), were extracted from 1 mL samples with N,N-dimethylformamide (DMF) according to the methods of Moran et al. [35]. After freezing at −18 °C for at least 24 h, the samples were centrifuged (10 min at 17,500 rcf), and the supernatant was analysed by measuring the absorption spectrum between 350 and 750 nm. Pigment concentrations were calculated via equations from Wellburn [36] and normalised to biomass (mg g_X_^−1^). Phycobiliproteins (PBPs) were extracted from 5 mL of sample concentrated in 1 mL of demineralised water and subjected to three cycles of freezing (−18 °C) and thawing (room temperature). After centrifugation (8 min at 13,200 rcf), the absorption spectrum (350–750 nm) of the supernatant was acquired. The concentrations of individual PBPs—phycocyanin (PC), allophycocyanin (APC), and phycoerythrin (PE)—were determined according to Bennett and Bogorad [37] and normalised to biomass (mg g_X_^−1^). The total PBP content was calculated as the sum of PC, APC, and PE. The biomass nitrogen content was analysed with a TOC-L instrument equipped with a TNM-L module (Shimadzu Corporation, Kyoto, Japan). One-millilitre samples were properly diluted with demineralised water to keep the amount of nitrogen within the calibration curve (5 µg L^−1^–100 mg L^−1^). The nitrogen quota (*q*_*N*_) was determined by the ratio of the measured nitrogen concentration to the biomass concentration in the sample and expressed as a percentage of dry weight (% DW). EPS production was quantified following the colorimetric method of Dubois et al. [38]. A 2 mL sample was centrifuged at 310 rcf for 25 min to separate and collect EPS in the supernatant while preserving cell integrity. For analysis, 200 µL of the supernatant was mixed with 200 µL of 5% (v/v) phenol and 1 mL of 95–98% (w/w) sulfuric acid. The mixture was maintained at room temperature for 10 min, followed by incubation at 30 °C for 15 min, before the absorbance was measured at 448 nm to quantify the glucose equivalent concentration in the sample, using as a reference a calibration curve prepared with D-glucose in the range of 10–150 µg L^−1^. All the analytical measurements were performed in duplicate for at least three biological replicates (n = 3).

### Iron quantification method

Iron quantification was performed via Agilent Technologies 7700 × ICP‒MS (Agilent Technologies International Japan, Ltd., Tokyo, Japan) following the ISO 17294–2:2016 procedure. The instrument was set specifically for trace element analysis by using the collision cell in “He” mode.

For the biomass samples, digestion was carried out by adding 0.5 g of nitric acid (HNO_3_, 69% w/w) to 0.5 g of the sample. The mixture was heated at 100 °C for at least 1 h to ensure complete digestion of the organic matrix and compound oxidation. The digested samples were then diluted with demineralised water to a final weight of 10 g, yielding a final HNO_3_ concentration of 3.5% (w/w). The residual debris was removed by filtration through a 0.22 µm filter. For undigested samples, 3 g of sample was mixed with 0.15 g of HNO_3_ (69% w/w) to achieve a consistent final HNO_3_ concentration of 3.5% (w/w) among all samples. Germanium (Ge) (TraceCERT®, 1000 mg L^−1^ in 2% HNO_3_, Sigma‒Aldrich) was employed as the internal standard and added to both the calibration and sample solutions at a concentration of 300 ng L^−1^. Instrument calibration was performed before each analysis via the IMS-120 (ULTRA SCIENTIFIC, lot CR-4190) standard across 12 points (10–100 ppb). Measurement quality control was performed in parallel via IV-ICPMS-71 (Inorganic Ventures), with recovery rates ranging from 95 to 102%.

In batch experiments, the iron content of the preinoculum (*q*_*Fe,0*_, mg_Fe_ kg_X_^−1^) was measured on digested biomass (*C*_*X,0*_). Iron consumption from the culture medium was assessed by determining the initial iron concentration in the medium (*C*_*Fe,0*_) and its residual concentration over time (*C*_*Fe,t*_). The iron content in the biomass at time *t* (*q*_*Fe,t*_) was calculated via Eq. (2) on the basis of the biomass concentration (*C*_*X,t*_). In continuous experiments, the total iron quota *q*_*Fe*_ was determined at the steady state on the basis of the iron concentration in the inlet medium (*C*_*Fe,in*_), the residual iron concentration in the outlet filtered samples (*C*_*Fe,out*_), and the biomass concentration (*C*_*X*_), as described in Eq. (3). The amount of iron adsorbed on the cell surface (*q*_*Fe,e*_) was determined via a protocol set up in this work: biomass samples containing 2 mg of dry weight were filtered (0.22 µm) and subjected to four sequential washes with 5 mL of 20 mM EDTA solution. The wash solutions were collected and analysed as previously described for undigested samples. During method development, it was verified that the iron mass balance closed across all measured fractions and that the washing protocol removed > 90% of surface-adsorbed iron. The internal iron content of the biomass (*q*_*Fe,i*_) was calculated according to Eq. (4). The adsorption ratio was calculated as the ratio between *q*_*Fe,e*_ and *q*_*Fe*_, expressed as a percentage of the latter (% *q*_*Fe*_).

2 $${q}_{Fe,t} = \frac{\left({q}_{Fe,0}\cdot {C}_{X,0}\right)+\left({C}_{Fe,0}-{C}_{Fe,t}\right)}{{C}_{X,t}}$$

3 $${q}_{Fe} = \frac{{C}_{Fe,in}-{C}_{Fe,out}}{{C}_{X}}$$

4 $${q}_{Fe,i} = {q}_{Fe}- {q}_{Fe,e}$$

## Results and discussion

### Rapid iron accumulation by *Nostoc* sp. cultivated in a HD batch system

*Nostoc* sp. was cultivated in a high-density (HD) system under diazo-phototrophic conditions with different iron concentrations in the medium following two different iron deprivation pretreatments. The latter aimed to reduce the intracellular iron content and observe growth differences on the basis of external substrate concentrations [39]. The tested iron concentrations were selected on the basis of literature data identifying the range of potentially growth-limiting iron levels [40] while also considering the specific cultivation system used. Compared with conventional systems, the HD cultivation system, in fact, results in significantly higher biomass concentrations [41], thus increasing the nutritional demand of microorganisms.

**Fig. 1:**
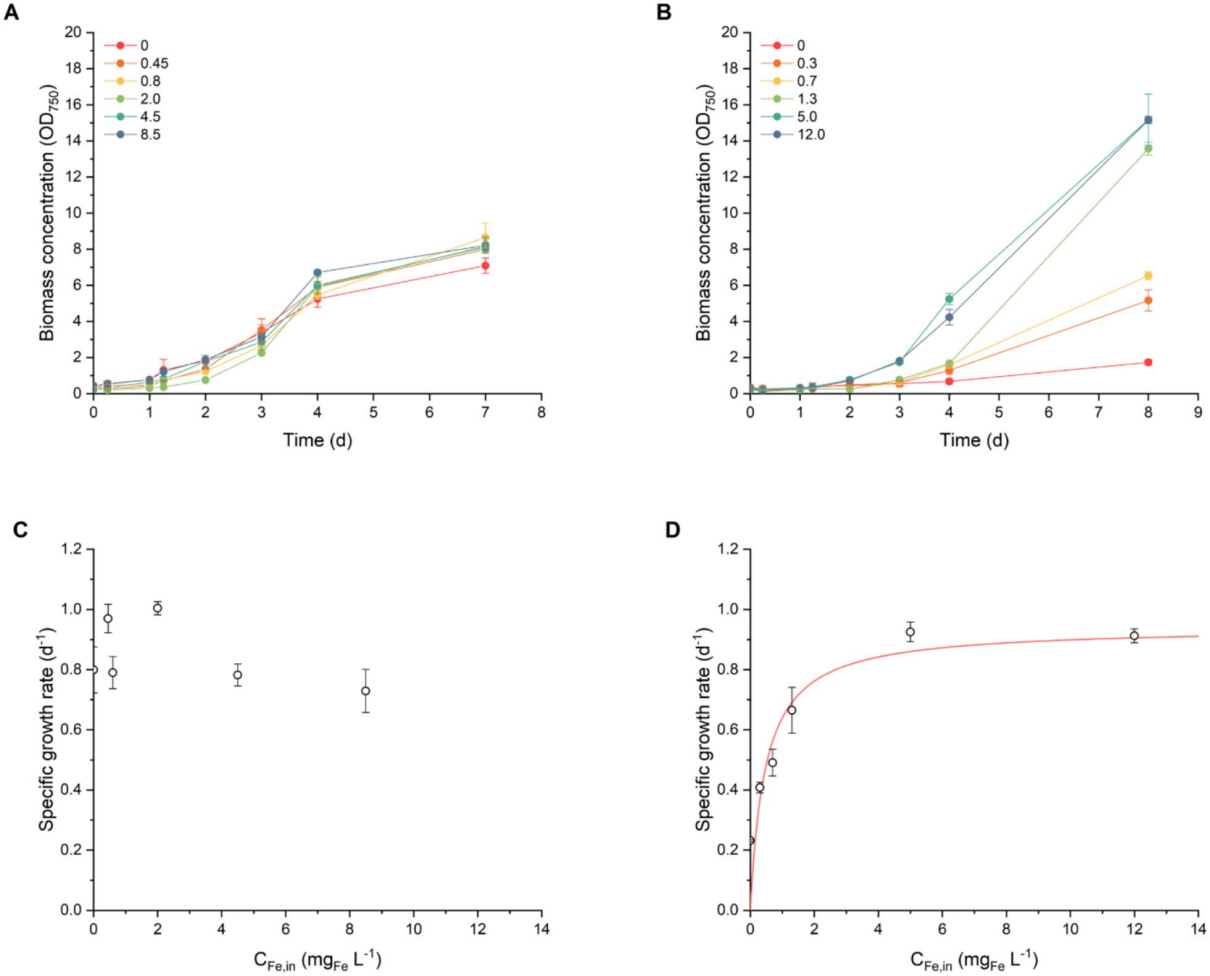
Growth curves (**A**–**B**) and maximum specific growth rates (*µ*, d^−1^) (**C**–**D**) at different initial iron concentration (*C*_*Fe,0*_, mg_Fe_ L^−1^) under diazo-phototrophic conditions following 2 days (left panels) and 10 days (right panels) of iron deprivation. Lines in panels A and B aid data visualisation, while panel D reports data fitting according to Eq. (1) (RSME = 0.10). Data are reported as the mean values, with error bars representing the standard deviation among replicates (n = 2)

Figure 1 (A) shows the growth curves under various initial iron concentrations (*C*_*Fe,0*_) following 2 days of iron deprivation. Across all the conditions, the growth trends were similar. A 2-day lag phase was observed, in line with previous findings in the HD system, due to the cultivation system and independent from the medium composition and iron content [33], followed by exponential growth transitioning to the stationary phase by day 4. By day 7, all the cultures had reached similar optical densities. *C*_*Fe,0*_ had no apparent effect on biomass growth, as comparable final concentrations were achieved even without iron supplementation. This might initially suggest that the tested concentrations were not low enough to induce iron limitation. However, this hypothesis can be excluded since *Nostoc* sp. sustained growth even in the absence of any iron supply to the medium, reaching 6.10 ± 0.04 g_X_ L^−1^ (Table 1). This behaviour points to the ability of microorganisms to rely on internal iron reserves to support short-term growth, even after 2 days of iron deprivation pretreatment. Similar trends have been reported in other cyanobacteria, such as *Spirulina*, where no evident differences in growth were observed between different iron concentrations up to 10 mg_Fe_ L^−1^ [26, 42, 43], suggesting that even the lowest iron concentration is sufficient to meet the physiological requirements for iron if the microorganism can efficiently regulate its internal reserves. Some studies report that reducing the iron content in cyanobacterial biomass requires long-term iron deprivation periods of up to 16 days [44, 45]. This prompted further investigation with an extended 10-day iron deprivation pretreatment, which revealed growth patterns influenced by *C*_*Fe,0*_, as shown in Fig. 1 (B). The growth increased proportionally with *C*_*Fe,0*_, up to 5.0 mg_Fe_ L^−1^, beyond which no marked differences were observed. At higher iron concentration, a 2-day lag phase was followed by rapid exponential growth. In contrast, at low or no iron availability, the exponential phase was less pronounced or nearly absent.

**Table 1:** Iron concentration in the medium (*C*_*Fe*_, mg_Fe_ L^−1^), biomass concentration (*C*_*X*_, g_X_ L^−1^), and iron quota (*q*_*Fe*_, mg_Fe_ kg_X_^−1^) over time (d) after 2 days and 10 days of iron deprivation under diazo-phototrophic conditions

Iron deprivation pretreatment: 2 days						
**Time** (d)	**C**_**Fe**_ (mg_Fe_ L^−1^)					
0	0.01 ± 0.00	0.45 ± 0.00	0.79 ± 0.00	1.96 ± 0.00	4.36 ± 0.13	8.32 ± 0.08
0.25	0.03 ± 0.00	0.07 ± 0.00	0.06 ± 0.00	0.12 ± 0.00	0.57 ± 0.03	1.74 ± 0.14
4	0.02 ± 0.00	0.06 ± 0.00	0.08 ± 0.00	0.20 ± 0.00	0.19 ± 0.05	0.16 ± 0.01
7	0.00 ± 0.00	0.01 ± 0.00	0.05 ± 0.00	0.12 ± 0.00	0.12 ± 0.04	0.21 ± 0.04
**Time** (d)	**C**_**X**_ (g_X_ L^−1^)					
0	0.52 ± 0.06	0.50 ± 0.04	0.58 ± 0.04	0.51 ± 0.04	0.52 ± 0.05	0.62 ± 0.05
0.25	0.52 ± 0.01	0.50 ± 0.05	0.58 ± 0.04	0.51 ± 0.04	0.52 ± 0.05	0.62 ± 0.05
4	3.46 ± 0.08	3.38 ± 0.07	3.48 ± 0.06	3.24 ± 0.05	3.43 ± 0.08	4.23 ± 0.05
7	6.10 ± 0.04	5.19 ± 0.04	5.90 ± 0.11	6.10 ± 0.05	6.70 ± 0.06	6.16 ± 0.08
**Time** (d)	**q**_**Fe**_ (mg_Fe_ kg_X_^−1^)					
0	212.5 ± 37.1	212.5 ± 37.1	212.5 ± 37.1	212.5 ± 37.1	212.5 ± 37.1	212.5 ± 37.1
0.25	174.9 ± 27.8	970.3 ± 106.8	1483 ± 131	3791 ± 348	7551 ± 1133	10,888 ± 1256
4	32.1 ± 4.8	145.5 ± 5.8	239.7 ± 7.0	577.2 ± 12.7	213.5 ± 86.0	967 ± 50
7	20.0 ± 2.3	104.9 ± 2.7	146.1 ± 4.2	319.7 ± 4.1	649.0 ± 32.2	1354 ± 38

**Iron deprivation pretreatment: 10 days**						
**Time** (d)	**C**_**Fe**_ (mg_Fe_ L^−1^)					
0	0.00 ± 0.00	0.30 ± 0.00	0.67 ± 0.00	1.27 ± 0.00	4.94 ± 0.07	11.62 ± 0.00
0.25	0.00 ± 0.00	0.15 ± 0.00	0.25 ± 0.00	0.14 ± 0.00	0.41 ± 0.09	0.84 ± 0.00
4	0.01 ± 0.00	0.00 ± 0.00	0.01 ± 0.00	0.01 ± 0.00	0.04 ± 0.01	0.26 ± 0.00
8	0.01 ± 0.01	0.02 ± 0.00	0.03 ± 0.00	0.05 ± 0.00	0.35 ± 0.33	0.22 ± 0.00
**Time** (d)	**C**_**X**_ (g_X_ L^−1^)					
0	0.56 ± 0.04	0.53 ± 0.04	0.51 ± 0.04	0.48 ± 0.04	0.53 ± 0.01	0.52 ± 0.04
0.25	0.56 ± 0.04	0.53 ± 0.04	0.51 ± 0.04	0.48 ± 0.04	0.53 ± 0.01	0.52 ± 0.05
4	0.71 ± 0.05	1.05 ± 0.13	1.40 ± 0.09	1.28 ± 0.06	2.26 ± 0.06	3.15 ± 0.05
8	1.20 ± 0.35	3.85 ± 0.07	4.70 ± 0.14	6.20 ± 0.16	7.40 ± 0.28	9.80 ± 0.22
**Time** (d)	**q**_**Fe**_ (mg_Fe_ kg_X_^−1^)					
0	74.7 ± 10.9	74.7 ± 10.9	74.7 ± 10.9	74.7 ± 10.9	74.7 ± 10.9	74.7 ± 10.9
0.25	83.9 ± 21.2	358.3 ± 31.7	883.7 ± 70.8	2428 ± 199	8656 ± 397	20,940 ± 2049
4	49.5 ± 2.3	324.9 ± 42.4	500.3 ± 33.6	1016 ± 51	2980 ± 280	3618 ± 58
8	12.1 ± 1.1	81.0 ± 2.2	141.6 ± 4.8	201.0 ± 5.78	658.1 ± 82.0	1166 ± 26

The data are reported as the means ± standard deviation among replicates (n = 2)

Consistent with the growth curves, iron availability did not markedly influence the specific growth rate (*µ*) after 2 days of iron deprivation (Fig. 1 (C)), with values ranging from 0.73 ± 0.07 d^−1^ to 1.00 ± 0.02 d^−1^ (Table S1), and no clear trends were observed. In contrast, the 10-day iron deprivation pretreatment revealed a distinct relationship: the growth rate increased rapidly with increasing *C*_*Fe,0*_ until reaching a threshold, beyond which further increases in iron availability no longer increased the growth rate, indicating saturation behaviour (Fig. 1 (D)). This trend reflects a typical nutrient-limited growth response, where the external substrate concentration becomes nonlimiting once the iron demands of microorganisms are met [46]. Interestingly, in iron-free medium, *Nostoc* sp. was again able to sustain growth in the short term, suggesting the exploitation of internal iron reserves. This evidence highlights the importance of intracellular nutrient storage and supports the relevance of kinetic models that incorporate internal quota dynamics, such as droop kinetics, over models that assume dependence on external availability [47]. Within this framework, the internal quota varies between a minimum threshold (*q*_*min*_), which is necessary for maintenance, and a physiological maximum (*q*_*max*_), which represents the limit of nutrient storage [48], and the half saturation constant for Fe uptake reasonably falls at approximately 0.5–0.8 mg_Fe_ L^−1^.

The iron quota in the preinoculum biomass (*q*_*Fe,0*_) was measured after the deprivation period. After 2 days of iron deprivation, the *q*_*Fe,0*_ was 212.5 ± 37.1 mg_Fe_ kg_X_^−1^ (Table 1). Although this value was consistent with the minimum quotas reported in the literature (i.e., 200 mg_Fe_ kg_X_^−1^) [9], it was not low enough to trigger clear iron limitation responses. Indeed, prolonged 10-day deprivation pretreatment resulted in a substantially lower initial quota of 74.7 ± 10.9 mg_Fe_ kg_X_^−1^ (Table 1), which enabled the observation of limited iron effects. These findings indicate that *Nostoc* sp. can tolerate and maintain growth at iron quotas well below the commonly referenced threshold, thereby broadening the known physiological range and highlighting the importance of internal iron dynamics in supporting growth under limiting conditions.

The observed behaviour is further supported by the dynamics of iron consumption from the culture medium and accumulation in biomass (*q*_*Fe*_) during early cultivation, as reported in supplementary Fig. S1 and S2. Unlike *C*_*Fe,0*_, *Nostoc* sp. rapidly removed nearly all available iron from the medium within the first 6 h of cultivation. Such rapid iron uptake has also been observed in *Spirulina*, where iron is quickly depleted from the medium and accumulates in biomass [26, 28]. In the present study, the reliability of iron consumption measurements was ensured through an analytical approach specifically designed to minimise artefacts related to iron precipitation or aggregation in solution. Filtration-based methods were employed instead of centrifugation, thereby accounting for potential colloidal iron forms, as noted by Kougia et al. [28]. Additionally, the EDTA concentration in the medium was carefully adjusted to maintain iron solubility while avoiding toxicity, which was confirmed by the consistency between the nominal and experimentally measured iron concentration. These precautions support the validity of the iron quota data and support the interpretation that *Nostoc* sp. exhibits “luxury uptake” of iron, storing excess amounts during early growth phases and gradually consuming them over time. Similar behavior has been reported for *Microcystis aeruginosa* [45].

**Fig. 2:**
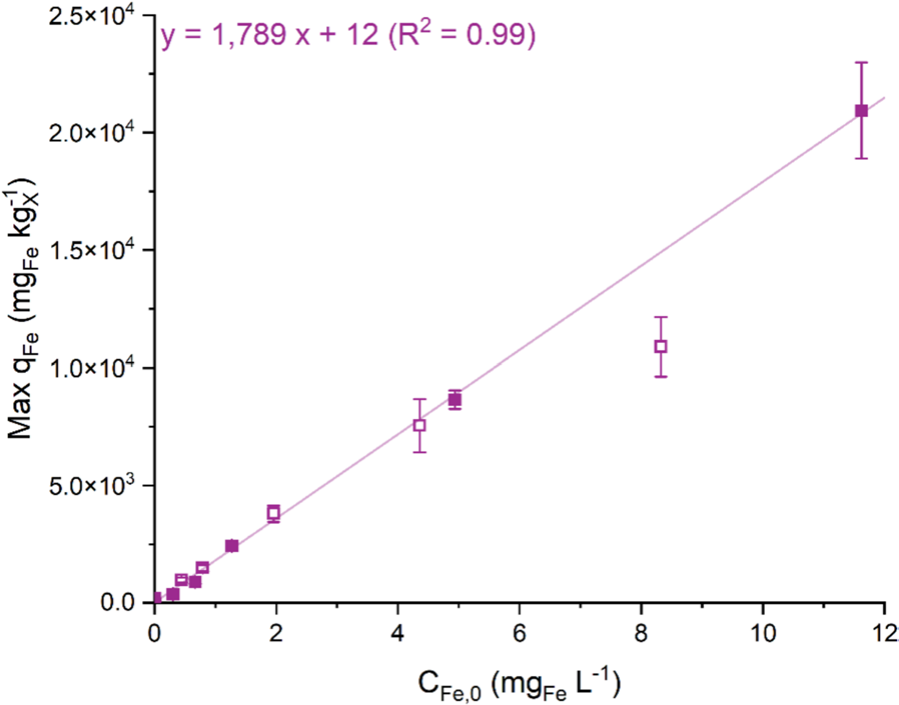
The highest iron quota (Max *q*_*Fe*_, mg_Fe_ kg_X_^−1^), measured after 6 h of cultivation, is reported as a function of the iron concentration in the medium (*C*_*Fe,0*_, mg_Fe_ L^−1^). The data are reported as the means, with error bars representing the standard deviation among replicates (n = 2). The solid symbols represent 2 days of iron deprivation; the solid symbols represent 10 days of iron deprivation

A linear relationship between *C*_*Fe,0*_ and the iron content in the biomass at its peak was observed (Fig. 2). Although very low values, such as 12 mg_Fe_ kg_X_^−1^, may result from approximate estimates of the viable cell fraction in the batch cultivation system and should therefore be interpreted with caution, the ability of *Nostoc* sp. to modulate the iron reserves in the biomass remains evident, with experimental values approaching 21,000 mg_Fe_ kg_X_^−1^ (Table 1). A similar relationship has been reported for *Arthrospira platensis*, where iron accumulation was also shown to scale proportionally with the iron concentration in the medium [26]. Interestingly, the observed trend was independent of the initial iron quota in the preinoculum (*q*_*Fe,0*_), as evidenced by all the data points aligning on the same regression line. This, together with the higher *q*_*Fe*_ values observed than the average values reported in the literature, suggests that not all measured iron may have been internalised. Biosorption has been reported in cyanobacteria and may explain the high q_Fe_ values observed [49]. Biosorption is a passive process involving metabolism-independent mechanisms (e.g., adsorption) and is distinct from bioaccumulation, which refers to an active process driven by metabolic pathways for internalising metals [50]. This aspect should be considered, as iron quotas may result from a combination of active uptake and surface adsorption. However, studying internal nutrient dynamics becomes challenging in batch systems, where transient phenomena such as adsorption often play a significant role.

### Biomass stress responses to iron limitation in continuous cultivation

**Fig. 3:**
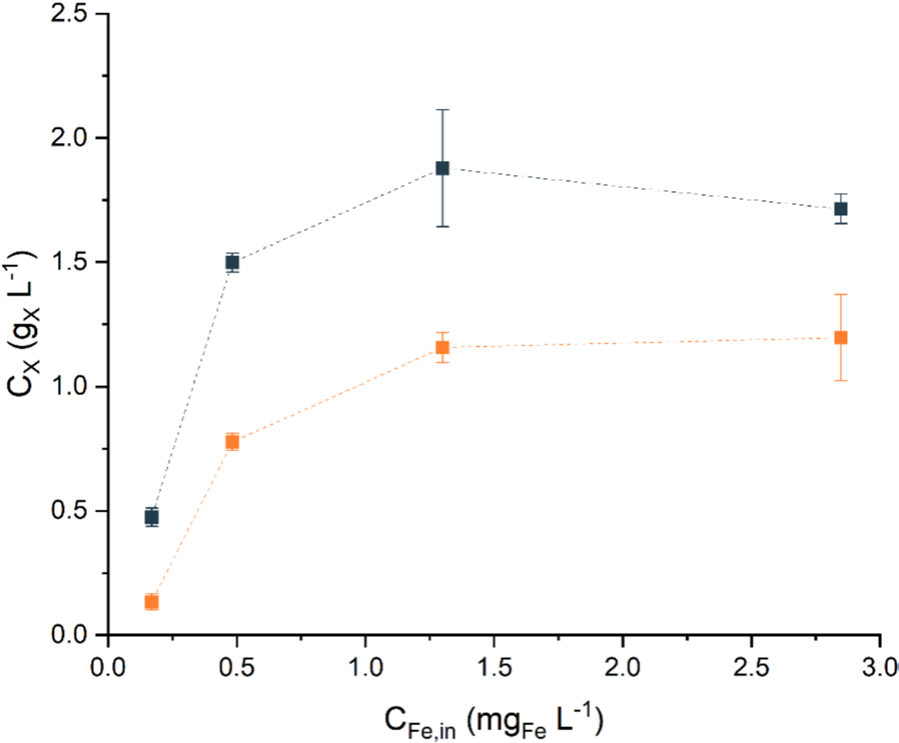
Steady-state biomass concentration (*C*_*X*_, g_X_ L^−1^) at different inlet iron concentrations (*C*_*Fe,in*_, mg_Fe_ L^−1^) and residence times (*τ*, d). Lines aid data visualisation. Data are reported as the mean values, with error bars representing the standard deviation among replicates (n = 4). Orange, *τ* = 1.2 ± 0.1 d; blue, *τ* = 2.2 ± 0.1 d

To better quantify the effect of the iron supply on the uptake yield, steady-state continuous reactors were used. The biomass concentration (*C*_*X*_) was monitored under steady-state conditions for various inlet iron concentrations (*C*_*Fe,in*_) at two residence times (*τ*), as shown in Fig. 3. At a residence time of 1.2 ± 0.1 d, *C*_*X*_ increased proportionally to iron availability at a lower *C*_*Fe,in*_ whereas saturation was observed above 1.3 mg_Fe_ L^−1^, which is reasonable given the value of the half-saturation constant retrieved from batch experiments. Similar saturation behaviour was observed at 2.2 ± 0.1 d, although *C*_*X*_ was consistently greater at each iron level than at the lower residence time. This difference can be attributed to the reduced dilution rates at longer residence times, which extend the retention time of cells within the PBR and allow for more efficient nutrient utilisation, enabling the biomass to accumulate at higher concentrations [51].

**Fig. 4:**
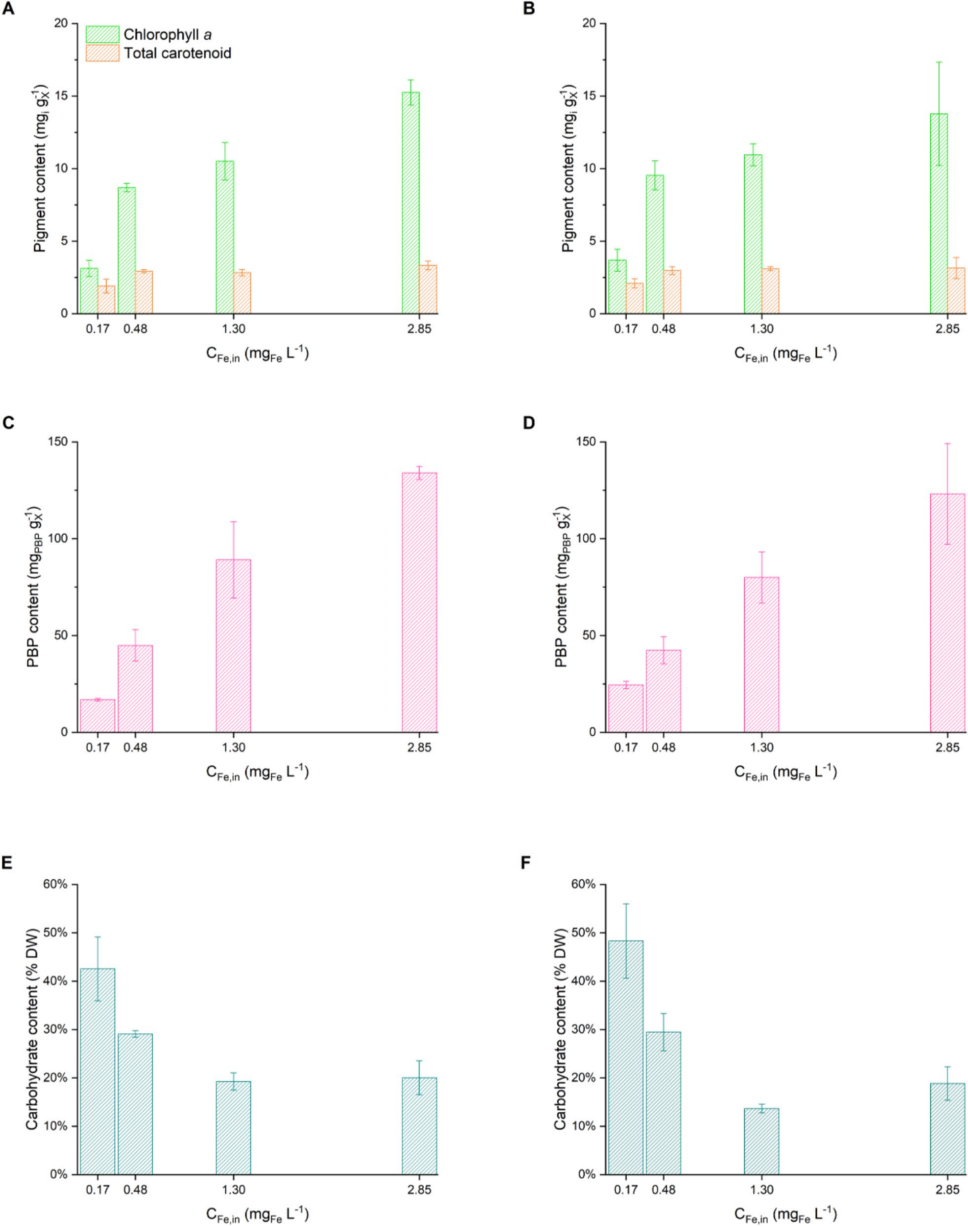
Steady-state contents of (**A**–**D**) pigments (mg_i_ g_X_^−1^) and (**E**–**F**) carbohydrates within the biomass (% DW) at different inlet iron concentration (*C*_*Fe,in*_, mg_Fe_ L^−1^) and residence times (*τ*, d). Data are reported as the mean values, with error bars representing the standard deviation among replicates (n = 4). (left panels) *τ* = 1.2 ± 0.1 d; (right panels) *τ* = 2.2 ± 0.1 d; green, chlorophyll *a*; orange, total carotenoids; pink, total phycobiliproteins; cyan, carbohydrates

Biomass composition was analysed to evaluate the response of microorganisms to different cultivation conditions (Fig. 4). The first clear effect of iron availability involves the photosynthetic apparatus, which includes Chl *a*, total carotenoids and phycobiliproteins. Under high iron availability (*C*_*Fe*,in_ = 2.85 ± 0.11 mg_Fe_ L^−1^), the Chl *a* and TC contents were similar to the physiological values for *Nostoc* sp. [52, 53], which were 15.2 ± 0.9 mg g_X_^−1^ and 3.3 ± 0.3 mg g_X_^−1^, respectively. In contrast, a greater limit (*C*_*Fe,in*_ = 0.17 ± 0.01 mg_Fe_ L^−1^) induced a strong decrease in Chl *a* (down to 3.1 ± 0.6 mg g_X_^−1^) along with a reduction in TC, which instead remained relatively stable at intermediate iron concentration (Table S2), given their dual role as light-harvesting pigments and protective agents for the photosynthetic apparatus [54]. This reduction aligns with the findings reported for iron-limited *Microcystis* sp. [55], *Chlorella pyrenoidosa* [56], and severely stressed *Synechocystis* sp. [57] and is translated visually into chlorosis due to the degradation of antenna complexes, a well-known phenomenon described for several cyanobacteria [26, 45, 58, 59]. Although iron is not a structural component of Chl*a*, it is directly involved in enzymatic pathways that are responsible for its synthesis [55], such as ferrochetalase, which catalyses key steps in the porphyrin biosynthesis pathway [60]. Similar trends were observed for PBP, whose content varied within a typical range [61] and increased with increasing iron availability, reaching a maximum of 134.0 ± 3.4 mg g_X_^−1^. The distributions of individual PBP categories, including phycocyanin (PC), allophycocyanin (APC), and phycoerythrin (PE), are shown in Fig. S3 and Table S3. Studies have demonstrated a direct relationship between increased iron availability and enhanced PBP biosynthesis, including specific components such as PCs and APCs [62]. Iron is not directly part of the PBP structure, but its availability influences the biosynthetic processes leading to phycobilins, the chromophores of PBPs [60].

The same behaviour was maintained for both residence times, with a slight increase in pigments at higher residence times, which was probably induced to compensate for the increased self-shading [63].

The impact of iron availability is not limited to pigment biosynthesis but also extends to other central aspects of cell metabolism. In addition to photosynthetic apparatus reorganisation, cyanobacteria are reported to activate adaptive strategies to optimise resource use and reduce iron demand, including the replacement of iron-dependent proteins with functional analogues [64–66]. As an example, in cyanobacteria, flavodoxin can replace ferredoxin, and non-iron-containing superoxide dismutase can substitute for iron-dependent forms [67]. These adaptations are also reported to affect nitrogen metabolism, which is often impaired under iron-limited stress conditions, with a negative effect on nitrogen fixation and heterocyst formation [18].

The nitrogen content (*q*_*N*_) of the biomass was therefore analysed to assess whether varying iron concentration influenced the ability of the microorganisms to fix and accumulate nitrogen. Unlike other variables, no meaningful effect of *C*_*Fe,in*_ on *q*_*N*_ was observed across residence times, as shown in Fig. S4. The nitrogen content varied within a narrow range of 7.3–9.3% DW across the tested conditions (Table S4), with no evident differences observed, except under iron-limiting conditions at a *C*_*Fe,in*_ of 0.17 ± 0.01 mg_Fe_ L^−1^ and a residence time of 2.2 ± 0.1 d, where the *q*_*N*_ decreased to 5.7 ± 0.2% DW. Despite the lower biomass concentrations achieved under iron-limiting conditions, the nitrogen content per unit biomass remained relatively stable, highlighting the strong ability of microorganisms to regulate nitrogen assimilation to maintain their cellular functionality. While less nitrogen was fixed overall due to the reduced biomass yield, *q*_*N*_ was largely conserved. Similar findings were previously highlighted, reporting that *q*_*N*_ in *Nostoc* sp. biomass cultivated in continuous systems was not strongly affected by varying conditions [68]. As an essential macronutrient fundamental to microbial metabolism, nitrogen is likely tightly regulated to maintain homeostatic levels, ensuring an adequate internal quota even under adverse environmental conditions. This regulation may be particularly evident in continuous systems, where maintaining steady-state conditions requires active cell replication.

The physiological state of the microorganisms was further characterised through the quantitative determination of carbohydrate content. The results are expressed as a percentage of dry weight (% DW) and reported in Fig. 4 (E–F) for both residence times. At relatively high iron concentration, the carbohydrate content, approximately 20% DW, remains at physiological levels [30]. However, as the iron concentration in the medium decreased, the carbohydrate content markedly increased, reaching maximum levels of 42.6 ± 6.6% DW and 48.3 ± 7.7% DW under the most limiting conditions (*C*_*Fe,in*_ = 0.17 ± 0.01 mg_Fe_ L^−1^) for residence times of 1.2 ± 0.1 d and 2.2 ± 0.1 d, respectively. The accumulation of carbohydrates is a common response in cyanobacteria under nutrient limitation. For example, *Arthrospira* sp. 8005 presented an increase in carbohydrate content under nitrogen-deprived conditions, ranging from 14 to 74% [69]. Similarly, *Synechocystis* sp., when exposed to iron limitation, exhibited a marked accumulation of carbohydrates and glycogen precursors, including glucose and other hexoses, highlighting a shift in metabolism toward carbon storage [70]. In the same study, iron deprivation was reported to induce responses analogous to those observed under nitrogen starvation, resulting from the close interplay between iron and nitrogen metabolism. The increase in carbohydrate content under iron-limiting conditions reflects a clear shift in metabolism towards the accumulation of carbon reserves, confirming the altered physiological state of the microorganism already suggested by the changes in the pigment profiles.

### Iron dynamics in continuous cultivation: the role of EPS production and iron adsorption

**Fig. 5:**
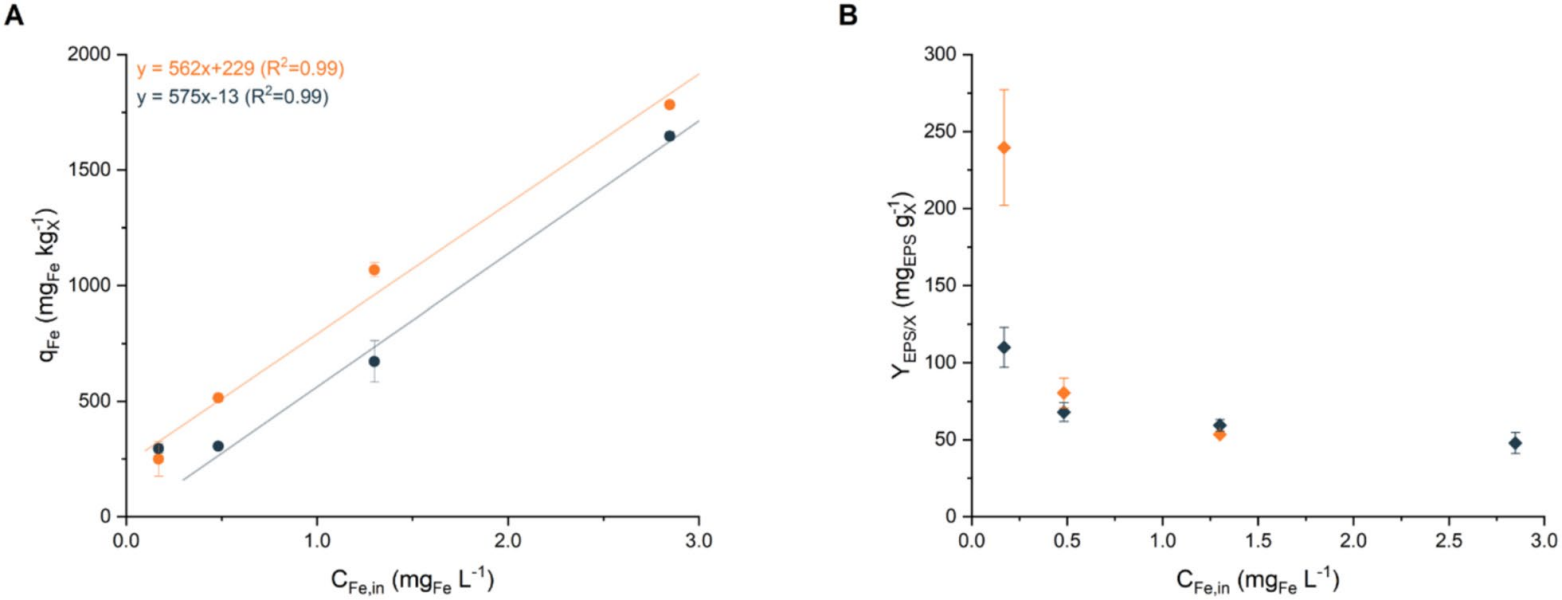
Steady-state (**A**) iron content (*q*_*Fe*_, mg_Fe_ kg_X_^−1^) and (**B**) EPS production (*Y*_*EPS/X*_, mg_EPS_ g_X_^−1^) at different inlet iron concentration (*C*_*Fe,in*_, mg_Fe_ L^−1^) and residence times (*τ*, d). Data are reported as the mean values, with error bars representing the standard deviation among replicates (n = 4). Orange, *τ* = 1.2 ± 0.1 d; blue, *τ* = 2.2 ± 0.1 d

To gain a deeper understanding of the observed responses and assess their relationships not only with external iron availability but also with the amount effectively accumulated by the microorganism, a quantitative analysis of iron content was performed. The total iron content in biomass (*q*_*Fe*_) was quantified under various inlet iron concentrations (*C*_*Fe,in*_), revealing a linear correlation between these two variables (R^2^ > 0.99) at both residence times (*τ*), as shown in Fig. 5. The regression slopes for the two datasets were similar, indicating that both datasets could be described by a single regression line with a vertical shift for a shorter residence time. This means that, at an equivalent *C*_*Fe,in*_, *q*_*Fe*_ is greater for shorter residence times, likely because of the higher growth rates, which may necessitate greater iron bioaccumulation to support rapid growth metabolism. However, this linear relationship breaks down at very low iron concentration (*C*_*Fe,in*_ = 0.17 ± 0.01 mg_Fe_ L^−1^). Under these conditions, *q*_*Fe*_ stabilised at approximately 250–295 mg_Fe_ kg_X_^−1^ for both residence times (Table S5), a value higher than those observed in batch cultivation and more aligned with the ranges reported in the literature [9]. This difference in the *q*_*Fe*_ values may be attributed to the different natures of the batch and continuous cultivation systems. In batch systems, the stationary growth phase often comprises a heterogeneous population of replicating and lysing cells, leading to a constant biomass concentration over time. The measured *q*_*Fe*_ in such systems represents an average, potentially influenced by iron released from lysed cells and subsequently used by actively growing cells. As a closed system, nonviable cells are not separated from the batch environment. This can result in *q*_*Fe*_ values significantly below 200 mg_Fe_ kg_X_^−1^, as observed in this study. However, it remains unclear whether such low iron quotas correspond to viable cells, as the batch system does not provide direct evidence to assess cell viability under these conditions. In contrast, under steady-state conditions, a homogeneous population of actively replicating cells is maintained [57], while nonreplicating or nonviable cells are continuously removed through dilution [71]. This ensures that the measured *q*_*Fe*_ effectively reflects only the iron content of actively dividing cells. Therefore, a higher minimum iron quota may be required to support continuous cell replication.

Despite similar *q*_*Fe*_ at low *C*_*Fe,in*_, differences in physiological stress responses and biomass concentrations were evident, as discussed earlier. Notably, only at very low iron availability was cellular aggregation observed, a phenomenon that is typically absent in the continuous cultivation of *Nostoc* sp. PCC 7120 within a CSTR system. This aggregation was an empirical, visually observed phenomenon and was not quantified experimentally, but it was noticeably different from the other conditions, where cultures appeared well mixed and suspended. This finding highlighted that severe iron limitation may trigger additional stress responses that could alter cell physiology and interactions. The analysis of EPS production revealed an additional physiological response to limiting iron concentration, complementing the previously observed changes in biomass composition, particularly regarding the increased carbohydrate content and pigment synthesis. Under most conditions, EPS production remained relatively stable (Fig. 5 (B)), ranging from 47.9 ± 6.9 mg_EPS_ g_X_^−1^ to 68.0 ± 6.1 mg_EPS_ g_X_^−1^, with no clear trends. However, a pronounced increase in EPS production was observed under severe iron limitation. At a residence time of 2.2 ± 0.1 d, EPS levels increased to 110.0 ± 13.0 mg_EPS_ g_X_^−1^, whereas at a shorter residence time of 1.2 ± 0.1 d, production nearly doubled to 239.6 ± 37.7 mg_EPS_ g_X_^−1^. The increased EPS production correlated with the pronounced biomass aggregation observed (not shown). While stable biomass concentration and composition confirmed steady-state conditions, the observed aggregation coincided with a strong reduction in photosynthetic pigment content (Fig. 4), indicative of an altered physiological state and possibly reduced cell viability. It cannot be excluded that, in the absence of EPS production, the combined effects of stronger iron limitation and high dilution rates could have resulted in biomass washout. In this sense, EPS production may play a role in facilitating cellular adhesion, enabling *Nostoc* sp. to form aggregates as a survival mechanism under severe iron limitation.

**Fig. 6:**
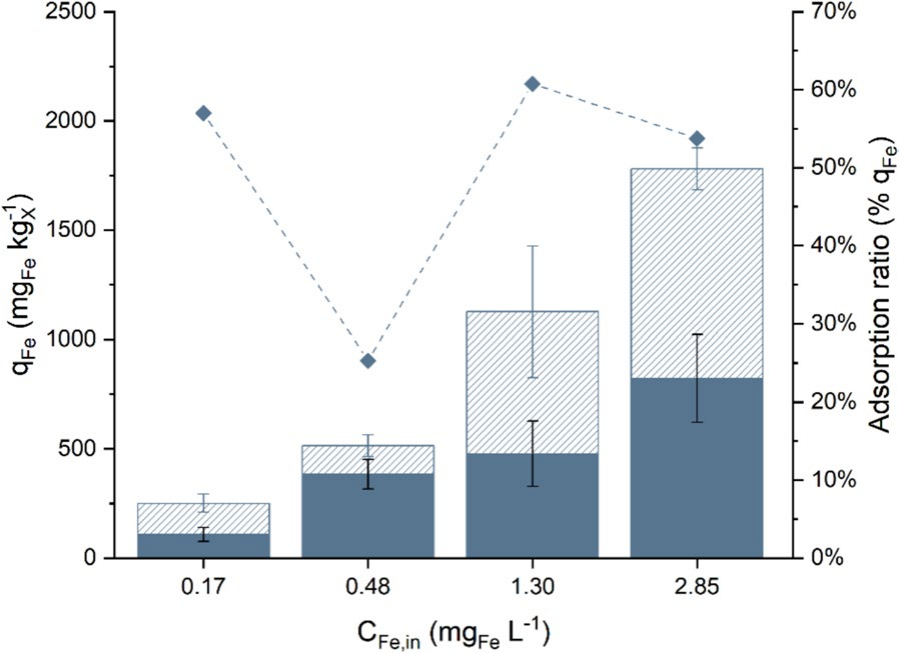
Steady-state iron distribution (bars) and adsorption ratio (diamonds) across the biomass at different inlet iron concentrations (*C*_*Fe,in*_, mg_Fe_ L^−1^) with a residence time of *τ* = 1.2 ± 0.1 d. Data are reported as the mean values, with error bars representing the standard deviation among replicates (n = 4). Filled bars, *q*_*Fe,i*_ (mg_Fe,i_ kg_X_^−1^); striped bars, *q*_*Fe,e*_ (mg_Fe,e_ kg_X_^−1^). Lines aid data visualisation

To better understand biomass behaviour with respect to EPS production under iron-limiting conditions, the iron content was further investigated to distinguish between the contents of intracellular iron (*q*_*Fe,i*_) and iron adsorbed on the biomass surface (*q*_*Fe,e*_) at shorter residence times (1.2 ± 0.1 d). The analysis revealed the presence of an adsorbed iron fraction under all tested conditions, demonstrating that adsorption phenomena can occur even in continuous cultivation systems at steady state (Fig. 6). *q*_*Fe,e*_ averaged 130–142 mg_Fe_ kg_X_^−1^ at low *C*_*Fe,in*_ values and increased to 649–959 mg_Fe_ kg_X_^−1^ when *C*_*Fe,in*_ exceeded 1.30 mg_Fe_ L^−1^ (Table S7). On the other hand, *q*_*Fe,i*_ stabilised at 385–477 mg_Fe_ kg_X_^−1^ under moderate iron availability (0.48–1.30 mg_Fe_ L^−1^), whereas strong iron depletion (0.17 ± 0.01 mg_Fe_ L^−1^) markedly reduced *q*_*Fe,i*_ to 108 ± 32 mg_Fe_ kg_X_^−1^. At *C*_*Fe,in*_ above 1.30 mg_Fe_ L^−1^, where no further increase in biomass was observed (Fig. 3), iron uptake increased, resulting in a higher intracellular iron quota of 821 ± 200 mg_Fe_ kg_X_^−1^. Figure 6 also displays the trend of the adsorption ratio (i.e., the fraction of adsorbed iron with respect to the total iron quota) as a function of *C*_*Fe,in*_. Between 1.30 and 2.85 mg_Fe_ L^−1^, indicating that the adsorption capacity was not saturated at 1.30 mg_Fe_ L^−1^ compared with 2.85 mg_Fe_ L^−1^. At 0.48 mg_Fe_ L^−1^, when growth was already negatively affected by the lower iron availability, the adsorption ratio was almost halved compared with the previous conditions. At the lowest tested concentration (0.17 mg_Fe_ L^−1^), the ratio increased again and stabilised at approximately 57%. A possible interpretation of this trend could be related to the increased production of EPS.

In accordance with experimental evidence, the literature reports increased production of EPS in response to iron limitation. EPS molecules, which are rich in negatively charged functional groups (such as carboxyl and hydroxyl groups), are able to bind cations, including ferric ions, in solution [72]. By storing iron externally, the microorganism creates a readily accessible reserve that can be mobilised when needed, helping to stabilise metabolism and sustain growth under adverse conditions. This mechanism could also explain the observed cell aggregation, as EPS mediate cell adhesion and act as a scaffold for iron adsorption, supporting survival in environments where intracellular accumulation may not be feasible.

In addition to EPS, the release of siderophores may also contribute to the accumulation of extracellular iron. Under conditions of iron scarcity, *Nostoc* sp. can secrete chelating molecules such as *schizokinen*, which can complex with high-affinity Fe^3+^ and facilitate its uptake via TonB-dependent transporters (TBDTs) [17]. Once inside, ferric iron is reduced to ferrous (Fe^2+^), becoming bioavailable for metabolic processes [73]. However, since this is an energy-consuming process [74], it is possible that under conditions of strong metabolic stress, these siderophores are secreted but not internalised. Additionally, iron homeostasis in *Nostoc* sp. is tightly regulated by the FurA repressor [75], which controls the expression of genes for uptake and storage, thus preventing toxicity related to pro-oxidant reactions [76]. To manage any intracellular surplus, cyanobacteria employ specialised iron-storage proteins, which sequester iron in a safe form, thus reducing its reactivity and providing a reserve [13]. In addition to intracellular storage, the absorption of iron on the cell surface could represent a buffering strategy to reduce oxidative stress while maintaining controlled external availability.

Taken together, these findings revealed a complex interplay between EPS production, iron adsorption, and intracellular iron regulation, highlighting the coordinated survival mechanisms employed by *Nostoc* sp. PCC 7120 to adapt to iron-limiting environments.

## Conclusions

This work demonstrated the remarkable physiological plasticity of *Nostoc* sp. PCC 7120 in coping with iron availability. The microorganisms presented highly variable iron contents, ranging from a few tens to tens of thousands of mg_Fe_ kg_X_^−1^, which were significantly broader than previously reported values and linearly proportional to the external iron concentration. A rapid accumulation of iron during the early stages of batch growth, followed by a gradual utilisation of accumulated reserves, was observed. However, the high measured values suggest a possibly consistent contribution of surface adsorption to total iron accumulation. Continuous experiments revealed a critical iron quota threshold below which active cell replication could not be sustained. This threshold likely reflects the minimum cellular iron requirement to meet metabolic demands and is influenced by the growth rate. Under low-iron availability conditions, a pronounced metabolic shift characterised by increased carbohydrate accumulation and a reduction in photosynthetic pigments was observed. In parallel, increased EPS production was recorded at the lowest tested iron concentration, which was associated with cell aggregation. Analysis of the distribution of iron between the intracellular environment and the cell surface, beyond confirming the occurrence of adsorption phenomena even under steady-state conditions, suggests that EPS production may represent a survival strategy aimed at maintaining the minimum total iron quota by creating a microniche around the cell, facilitating interactions with iron ions and forming a readily accessible reserve.

## Supplementary Information

Additional file 1. Supplementary tablesand figuresreporting growth kinetics, pigment and nitrogen composition, iron distribution, and EPS production of *Nostoc* sp. PCC 7120 under different iron concentration and residence time.

## Data Availability

The datasets used and/or analysed during the current study are available from the corresponding author on reasonable request.

## Abbreviations

ROS: Reactive oxygen species
PSI/II: Photosystem I/II
SOD: Superoxide dismutase
PAR: Photosynthetically active radiation
HD: High density
DoE: Design of experiments
PBR: Photobioreactor
CSTR: Continuous stirred-tank reactor
HRT: Hydraulic retention time
EPS: Exopolysaccharides
PFD: Photon flux density (µmol m^−2^ s^−1^)
OD: Optical density
DW: Dry weight
Chl*a*: Chlorophyll *a*
TC: Total carotenoids
PBP: Phycobiliproteins
PC: Phycocyanin
APC: Allophycocyanin
PE: Phycoerythrin
*τ*: Residence time (d)
*C*_*X*_: Biomass concentration (g_X_ L^−1^)
*C*_*Fe*_: Iron concentration (mg_Fe_ L^−1^)
*µ*: Growth rate (d^−1^)
*q*_*N*_: Nitrogen quota (%DW)
*q*_*Fe*_: Iron quota (mg_Fe_ kgX^−1^)
*q*_*Fe,e*_: Adsorbed iron quota (mg_Fe_ kg_X_^−1^)
*q*_*Fe,i*_: Intracellular iron quota (mg_Fe_ kg_X_^−1^)
*Y*_*EPS/X*_: EPS production (mg_EP__S_ g_X_^−1^)
*t*: Time (d)
