# Supplementary material for: Iron accumulation dynamics in Nostoc sp. PCC 7120: extent of biosorption in batch and continuous reactors
Source: Biotechnol Biofuels Bioprod. 2026 Jan 28;19:22. doi: 10.1186/s13068-026-02743-9 (PMC12931063; doi:10.1186/s13068-026-02743-9)
Supplement: Supplementary file 1 — Additional file 1. Supplementary tablesand figuresreporting growth kinetics, pigment and nitrogen composition, iron distribution, and EPS production of Nostoc sp. PCC 7120 under different iron concentration and residence time. [file 13068_2026_2743_MOESM1_ESM.docx]

**Supporting information**

for

**Iron accumulation dynamics in *Nostoc* sp. PCC 7120: extent of biosorption in batch and continuous reactors**

**Table S1.** Specific growth rates (*µ*, d^-1^) at varying iron concentrations in the medium (*C_Fe,0_*, mg_Fe_ L^-1^), and iron deprivation pre-treatments. Data are reported as mean values ± variability among replicates (n = 2).

| **Iron deprivation** | 2-day | **Iron deprivation** | 10-day |
| --- | --- | --- | --- |
| **C_Fe,0_** (mg_Fe_ L^-1^) | **µ** (d^-1^) | **C_Fe,0_** (mg_Fe_ L^-1^) | **µ** (d^-1^) |
| 0.01 ± 0.00 | 0.80 ± 0.08 | 0.00 ± 0.00 | 0.23 ± 0.00 |
| 0.45 ± 0.00 | 0.97 ± 0.05 | 0.30 ± 0.00 | 0.41 ± 0.02 |
| 0.79 ± 0.00 | 0.79 ± 0.05 | 0.67 ± 0.00 | 0.49 ± 0.04 |
| 1.96 ± 0.00 | 1.00 ± 0.02 | 1.27 ± 0.00 | 0.67 ± 0.08 |
| 4.36 ± 0.13 | 0.78 ± 0.04 | 4.94 ± 0.00 | 0.93 ± 0.03 |
| 8.32 ± 0.08 | 0.73 ± 0.07 | 11.62 ± 0.00 | 0.91 ± 0.02 |

**Table S2.** Steady-state pigment content within the biomass (mg_i_ g_X_^-1^) at different inlet iron concentrations (*C_Fe_*_,in_, mg_Fe_ L^-1^) and residence times (*τ*, d). Data are reported as mean values ± variability among replicates (n = 2). *Chla*, chlorophyll *a*; *TC*, total carotenoids.

| **Residence time** (d) | 1.2 ± 0.1 | | 2.2 ± 0.1 | |
| --- | --- | --- | --- | --- |
| **C_Fe,in_** | **Pigment content** (mg_i_ g_X_^-1^) | | | |
| (mg_Fe_ L^-1^) | *Chla* | *TC* | *Chla* | *Car* |
| 0.17 ± 0.01 | 3.1 ± 0.6 | 1.9 ± 0.5 | 3.7 ± 0.7 | 2.1 ± 0.3 |
| 0.48 ± 0.03 | 8.7 ± 0.3 | 2.9 ± 0.1 | 9.5 ± 1.0 | 3.0 ± 0.3 |
| 1.30 ± 0.04 | 10.5 ± 1.3 | 2.8 ± 0.2 | 10.9 ± 0.8 | 3.1 ± 0.1 |
| 2.85 ± 0.11 | 15.2 ± 0.9 | 3.3 ± 0.3 | 13.8 ± 3.6 | 3.2 ± 0.7 |

**Table S3.** Steady-state phycobiliprotein content within the biomass (mg_i_ g_X_^-1^) at different inlet iron concentrations (*C_Fe_*_,in_, mg_Fe_ L^-1^) and residence times (*τ*, d). Data are reported as mean values ± variability among replicates (n = 2). *PBP*, total phycobiliproteins; *PC*, phycocyanin; *APC*, allophycocyanin; *PE*, phycoerythrin.

| **Residence time** (d) | 1.2 ± 0.1 | | | |
| --- | --- | --- | --- | --- |
| **C_Fe,in_** | **Pigment content** (mg_i_ g_X_^-1^) | | | |
| (mg_Fe_ L^-1^) | *PBP* | *PC* | *APC* | *PE* |
| 0.17 ± 0.01 | 16.9 ± 0.7 | 10.9 ± 0.2 | 3.5 ± 0.1 | 2.5 ± 0.4 |
| 0.48 ± 0.03 | 44.9 ± 8.1 | 35.6 ± 6.4 | 9.3 ± 1.7 | 0.0 ± 0.0^a^ |
| 1.30 ± 0.04 | 71.3 ± 19.7 | 71.3 ± 11.4 | 17.8 ± 7.6 | 0.0 ± 0.0^a^ |
| 2.85 ± 0.11 | 134.0 ± 3.4 | 89.9 ± 7.7 | 37.3 ± 4.8 | 6.7 ± 1.0 |
| **Residence time** (d) | 2.2 ± 0.1 | | | |
| **C_Fe,in_** | **Pigment content** (mg_i_ g_X_^-1^) | | | |
| (mg_Fe_ L^-1^) | *PBP* | *PC* | *PBP* | *PC* |
| 0.17 ± 0.01 | 24.5 ± 1.9 | 14.6 ± 0.5 | 5.6 ± 1.1 | 4.4 ± 1.1 |
| 0.48 ± 0.03 | 42.4 ± 7.1 | 35.5 ± 0.4 | 7.0 ± 0.4 | 0.0 ± 0.0^a^ |
| 1.30 ± 0.04 | 80.0 ± 13.2 | 60.9 ± 10.9 | 19.1 ± 4.9 | 0.0 ± 0.0^a^ |
| 2.85 ± 0.11 | 123.1 ± 29.0 | 86.3 ± 15.1 | 36.8 ± 14.3 | 0.0 ± 0.0^a^ |


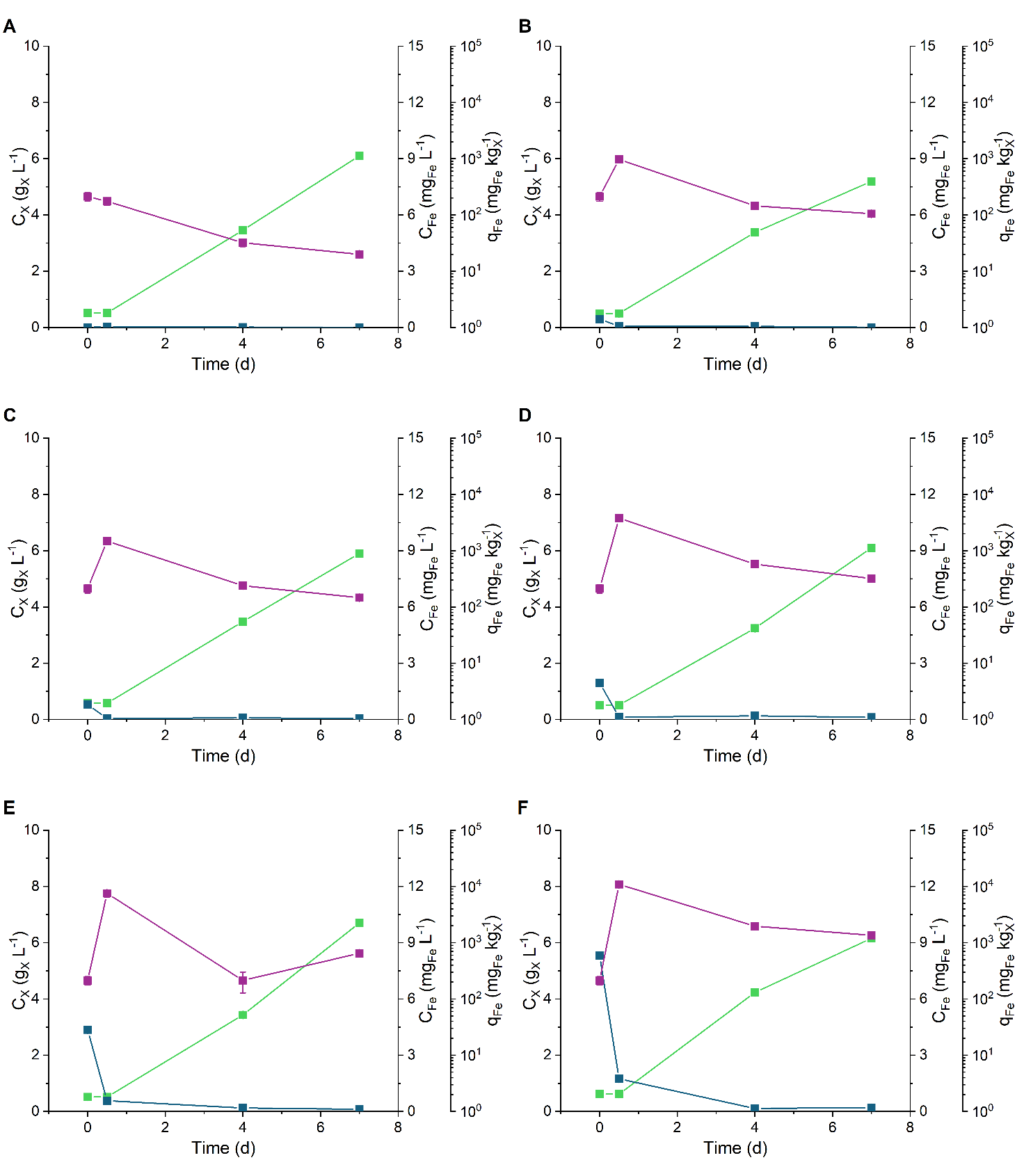


**Fig. S1** Biomass concentration (*C_X_*, green), iron concentration in the medium (*C_Fe_*, blue), and iron quota (*q_Fe_,* purple) over time (d) after 2 days of iron deprivation under diazo-phototrophic conditions. Each panel corresponds to a different initial *C_Fe_*. Data are reported as mean values, with error bars representing the variability among replicates (n = 2).


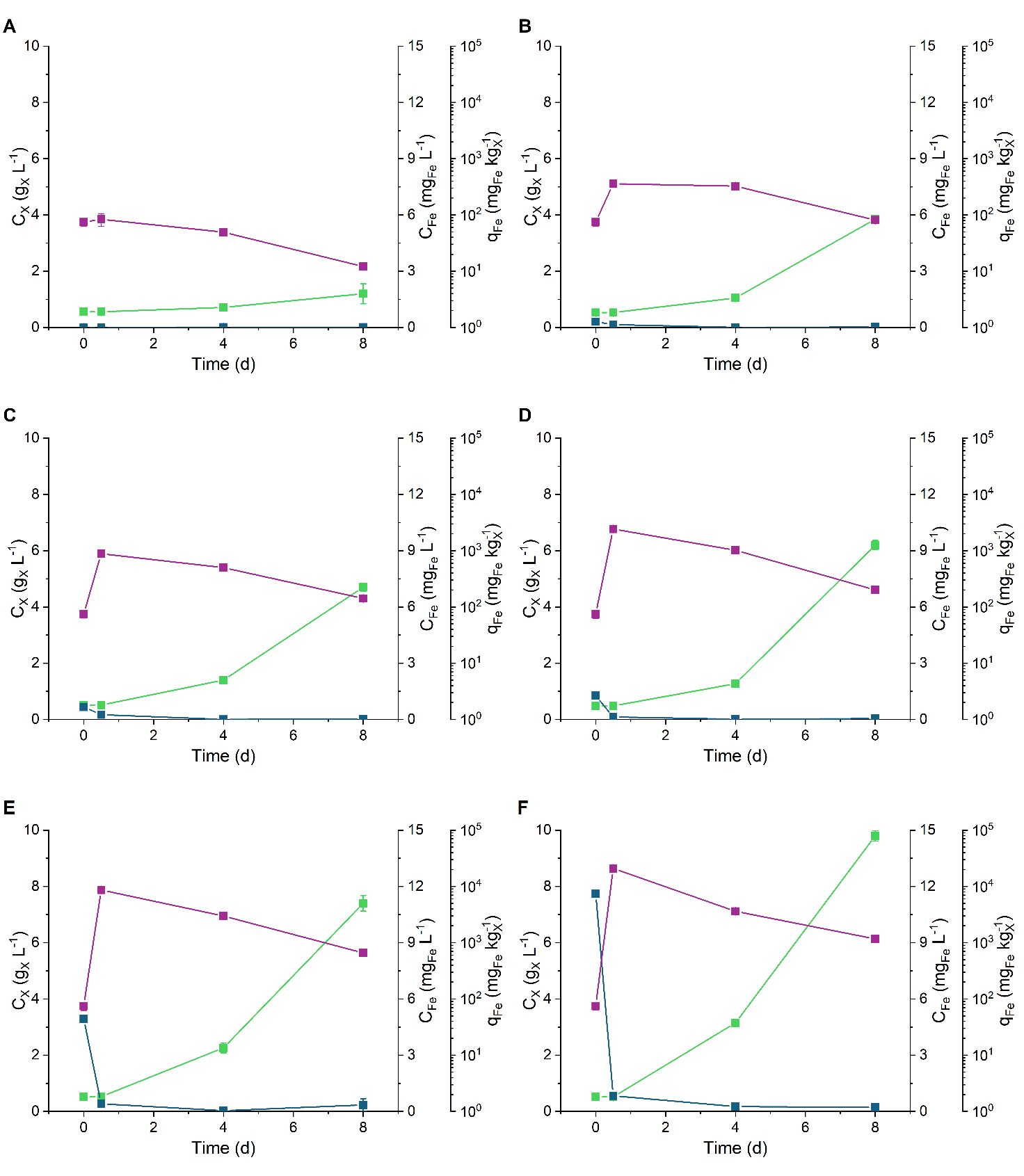


**Fig. S2** Biomass concentration (*C_X_*, green), iron concentration in the medium (*C_Fe_*, blue), and iron quota (*q_Fe_*, purple) over time (d) after 10 days of iron deprivation under diazo-phototrophic conditions. Each panel corresponds to a different initial *C_Fe_*. Data are reported as mean values, with error bars representing the variability among replicates (n = 2).


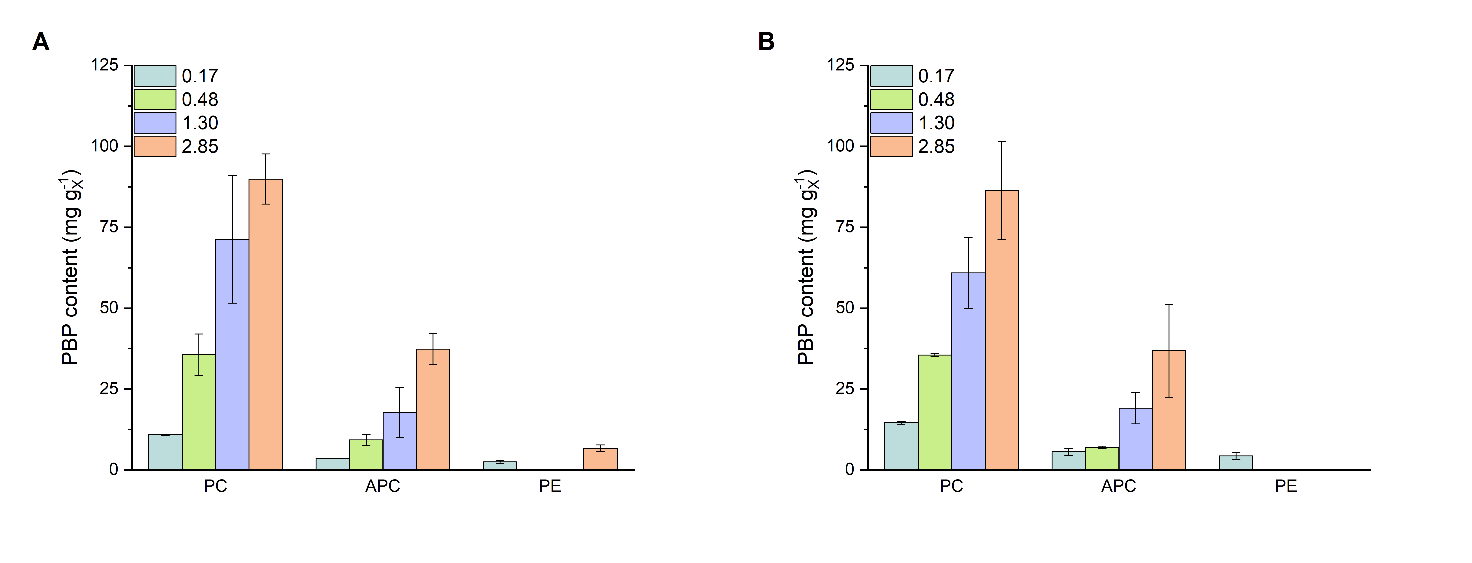


**Fig. S3** Steady-state phycobiliprotein content within the biomass (mg_i_ g_X_^-1^) at different inlet iron concentrations (*C_Fe,in_*, mg_Fe_ L^-1^) and residence times (*τ*, d). Data are reported as mean values, with error bars representing the variability among replicates (n = 4). (**A**) *τ* = 1.2 ± 0.1 d; (**B**) *τ* = 2.2 ± 0.1 d. *PC*, phycocyanin; *APC*, allophycocyanin; *PE*, phycoerythrin.


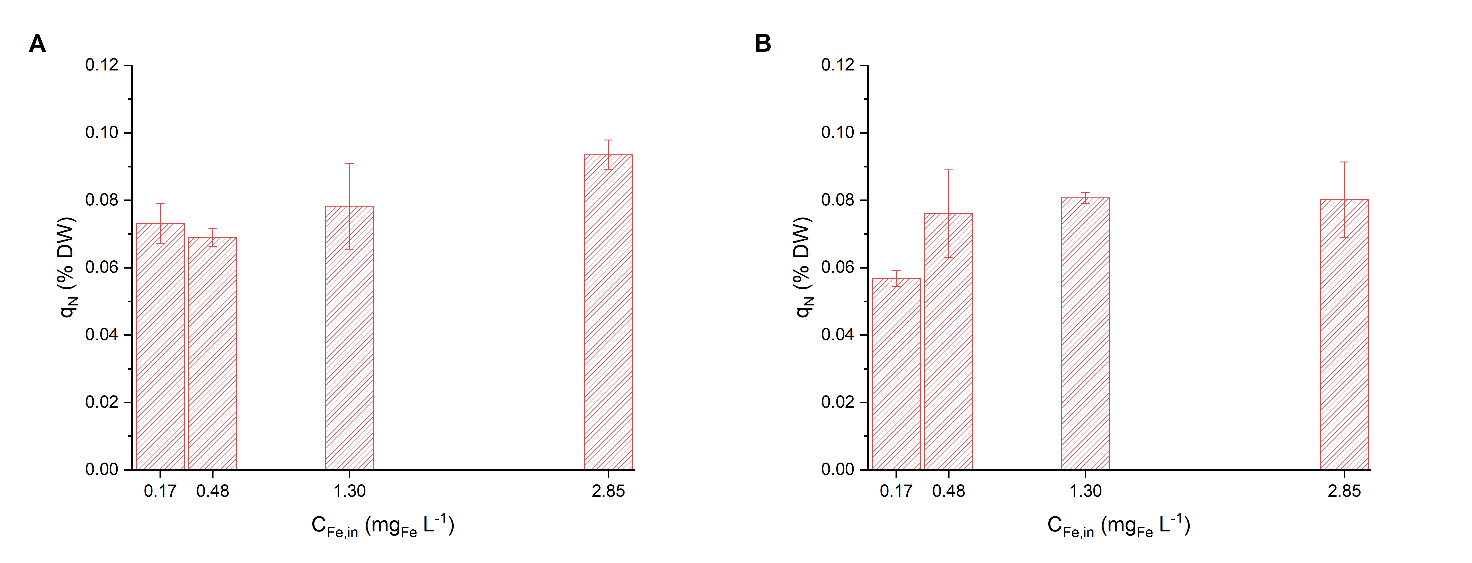


**Fig. S4** Steady-state nitrogen content within the biomass (*q_N_*, % DW) at different inlet iron concentrations (*C_Fe,in_*, mg_Fe_ L^-1^) and residence times (*τ*, d). Data are reported as mean values, with error bars representing the variability among replicates (n = 4). (**A**) *τ* = 1.2 ± 0.1 d; (**B**) *τ* = 2.2 ± 0.1 d.

**Table S4.** Steady-state nitrogen content within the biomass (*q_N_*, % DW) at different inlet iron concentrations (*C_Fe,in_*, mg_Fe_ L^-1^) and residence times (*τ*, d). Data are reported as mean values ± variability among replicates (n = 4).

| **Residence time** (d) | 1.2 ± 0.1 | 2.2 ± 0.1 |
| --- | --- | --- |
| **C_Fe,in_** (mg_Fe_ L^-1^) | **q_N_** (% DW) | |
| 0.17 ± 0.01 | 7.3 ± 0.6 | 5.7 ± 0.2 |
| 0.48 ± 0.03 | 6.9 ± 0.3 | 7.6 ± 1.3 |
| 1.30 ± 0.04 | 7.8 ± 1.3 | 8.1 ± 0.2 |
| 2.85 ± 0.11 | 9.3 ± 0.4 | 8.0 ± 1.1 |

**Table S5.** Steady-state total iron content (*q_Fe_*, mg_Fe_ kg_X_^-1^) at different inlet iron concentrations (*C_Fe,in_*, mg_Fe_ L^-1^) and residence times (*τ*, d). Data are reported as mean values ± variability among replicates (n = 4).

| **Residence time** (d) | 1.2 ± 0.1 | 2.2 ± 0.1 |
| --- | --- | --- |
| **C_Fe,in_** (mg_Fe_ L^-1^) | **q_Fe_** (mg_Fe_ kg_X_^-1^) | |
| 0.17 ± 0.01 | 250 ± 74 | 295 ± 19 |
| 0.48 ± 0.03 | 515 ± 17 | 306 ± 12 |
| 1.30 ± 0.04 | 1068 ± 32 | 672 ± 90 |
| 2.85 ± 0.11 | 1783 ± 3 | 1647 ± 18 |

**Table S6.** Steady-state EPS production (*Y_EPS/X_*, mg_EPS_ g_X_^-1^) at different inlet iron concentrations (*C_Fe,in_*, mg_Fe_ L^-1^) and residence times (*τ*, d). Data are reported as mean values ± variability among replicates (n = 4).

| **Residence time** (d) | 1.2 ± 0.1 | 2.2 ± 0.1 |
| --- | --- | --- |
| **C_Fe,in_** (mg_Fe_ L^-1^) | **Y_EPS/X_** (mg_EPS_ g_X_^-1^) | |
| 0.17 ± 0.01 | 239.6 ± 37.7 | 110.0 ± 13.0 |
| 0.48 ± 0.03 | 80.4 ± 9.7 | 68.0 ± 6.1 |
| 1.30 ± 0.04 | 53.4 ± 1.0 | 59.4 ± 3.9 |
| 2.85 ± 0.11 |  | 47.9 ± 6.9 |

**Table S7.** Steady-state iron distribution among intracellular quota (*q_Fe,i_*, mg_Fe_ kg_X_^-1^) and adsorbed quota (*q_Fe,e_*, mg_Fe_ kg_X_^-1^) at different inlet iron concentrations (*C_Fe,in_*, mg_Fe_ L^-1^) and a residence time (*τ*) of 1.2 ± 0.1 d, along with adsorption efficiency (% *q_Fe_*). Data are reported as mean values ± variability among replicates (n = 4).

| **Residence time** (d) | 1.2 ± 0.1 | | | |
| --- | --- | --- | --- | --- |
| **C_Fe,in_** (mg_Fe_ L^-1^) | 0.17 ± 0.01 | 0.48 ± 0.03 | 1.30 ± 0.04 | 2.85 ± 0.11 |
| **q_Fe,i_** (mg_Fe_ kg_X_^-1^) | 108 ± 32 | 385 ± 68 | 477 ± 150 | 821 ± 200 |
| **q_Fe,e_** (mg_Fe_ kg_X_^-1^) | 142 ± 42 | 130 ± 50 | 649 ± 300 | 959 ± 96 |
| **Ads. efficiency** (% q_Fe_) | 57.0% | 25.3% | 60.8% | 53.8% |
